# Effects of CO_2_ Enrichment on Growth and Development of *Impatiens hawkeri*

**DOI:** 10.1100/2012/601263

**Published:** 2012-03-12

**Authors:** Fan-Fan Zhang, Yan-Li Wang, Zhi-Zhe Huang, Xiao-Chen Zhu, Feng-Jiao Zhang, Fa-Di Chen, Wei-Min Fang, Nian-Jun Teng

**Affiliations:** College of Horticulture, Nanjing Agricultural University, Nanjing 210095, China

## Abstract

The effects of CO_2_ enrichment on growth and development of *Impatiens hawkeri*, an important greenhouse flower, were investigated for the purpose of providing scientific basis for CO_2_ enrichment to this species in greenhouse. The plants were grown in CO_2_-controlled growth chambers with 380 (the control) and 760 (CO_2_ enrichment) *μ*mol·mol^−1^, respectively. The changes in morphology, physiology, biochemistry, and leaf ultrastructure of *Impatiens* were examined. Results showed that CO_2_ enrichment increased flower number and relative leaf area compared with the control. In addition, CO_2_ enrichment significantly enhanced photosynthetic rate, contents of soluble sugars and starch, activities of peroxidase (POD), superoxide dismutase (SOD), and ascorbate peroxidase (APX), but reduced chlorophyll content and malondialdehyde (MDA) content. Furthermore, significant changes in chloroplast ultrastructure were observed at CO_2_ enrichment: an increased number of starch grains with an expanded size, and an increased ratio of stroma thylakoid to grana thylakoid. These results suggest that CO_2_ enrichment had positive effects on *Impatiens*, that is, it can improve the visual value, promote growth and development, and enhance antioxidant capacity.

## 1. Introduction

Atmospheric concentrations of greenhouse gases such as CO_2_, N_2_O, and CH_4_ are increasing quickly since the beginning of the industrial revolution, which results in a rise in ground-level air temperatures [1–4]. These global climate changes will have produced profound effects on plant physiology and growth, structure and function of plant populations, and species distributions [2–4]. Among these factors, CO_2_ is one of the raw materials of photosynthesis and has great influences on plant growth and development. The current atmospheric CO_2_ concentration is about 380 *μ*mol*·*mol^−1^, which is far below the optimum concentration of plant photosynthesis [5], especially for those plants such as ornamentals grown in greenhouse where ventilation is so limited to supplement the CO_2_ consumed by plant photosynthesis, thus seriously affecting the growth, development, yield, and visual value of greenhouse-grown ornamentals [6, 7].

Over the past three decades, a large number of studies have focused on the effects of CO_2_ enrichment (elevated CO_2_) on the growth and development of plants. Generally, plants grown at elevated CO_2_ relative to those grown at ambient CO_2_ often exhibit increased growth and photosynthesis, lower transpiration, inhibited respiration, improved water use efficiency, decreased mineral nutrient concentrations, increased plant hormones contents, reduced stomatal density and conductance, and so forth [8–11]. However, most of these studies are focused on trees, steppe plants, crop plants, and greenhouse-grown vegetables, but substantial knowledge about potential influences of CO_2_ enrichment on greenhouse-grown ornamentals is lacking [12]. Besides, available studies on greenhouse-grown ornamentals are usually focused on the morphology, photosynthesis, yield, and visual value of plants [7], few of them have investigated the impacts of CO_2_ enrichment on antioxidant enzyme system and leaf ultrastructure, which are very important for an integrative understanding of plant responses to elevated CO_2_.

*Impatiens hawkeri or Impatiens* New Guinea is a perennial species with rich colors and long flowering period, which has become a popular and important potted flowering plant throughout the world in recent years [13]. According to our knowledge, a study concentrating on the responses of* Impatiens* New Guinea to CO_2_ enrichment has not been reported until now. Therefore, to better understand the effects of CO_2_ enrichment on growth and development of *Impatiens* New Guinea, we investigated the influences of CO_2_ enrichment on its morphological characters, photosynthetic rate, chlorophyll content, nonstructural carbohydrates content, in particular antioxidant enzyme activity, and leaf ultrastructure. Although the results in the artificial greenhouse environment are possibly different from the results expected in the real world because of the nonlinear nature of the CO_2_ concentrations and the temporal and spatial variability of the CO_2_ concentration in the filed observations [1–3], our study will provide useful knowledge for supplementing CO_2_ during growth of ornamentals and even other horticultural crops in greenhouse.

## 2. Materials and Methods

### 2.1. Plant Materials and Growth Conditions

Seedlings of *Impatiens* were bought from a local company (Changshu Agricultural Technology Development Co., Ltd.) and rooted in 200 cm^3^ plastic pots filled with medium consisting of a 2 : 1 [volume/volume (v/v)] mixture of peat and vermiculite. After that, plants were cultivated for ten weeks in CO_2_-controlled growth chambers which can automatically and accurately control environmental factors including temperature, light, relative humidity, photosynthetically active radiation (PAR), and CO_2_ concentration according to preset data. The plants were alternately watered to saturation with Murashige-Skoog (MS) solution or deionized water. During the first four weeks, plants were fertilized to saturation with 1/3 MS solution every two weeks and with 1/2 MS solution every two weeks in the following six weeks. Plants were grown under a 12 h photoperiod with 300 *μ*mol*·*m^−2^ *·*s^−1^ PAR and day : night temperatures of 25 : 18°C. The relative humidities during daytime and at time were maintained at 70–80% and over 90%, respectively. Control plants were grown in one CO_2_-controlled growth chamber with CO_2_ concentration of 380 ± 30 *μ*mol*·*mol^−1^, while the treated plants were grown in another one with CO_2_ concentration of 760 ± 50 *μ*mol*·*mol^−1^ during daytime and 380 ± 30 *μ*mol*·*mol^−1^ at night. The purity of CO_2_ applied in this study was 99.999%. Except for CO_2_ concentration of CO_2_, other environmental conditions in two growth chambers were common. In order to keep the potential for interactive effects between the chambers and the developmental stage of the plants to a minimum, the CO_2_ concentrations of the two chambers were swapped, and the pots were moved between chambers and randomly rearranged weekly. This purpose is to average out any possible effects from the chambers and pot positions within the chambers.

### 2.2. Growth and Development Analysis

After ten weeks' cultivation under either 380 *μ*mol*·*mol^−1^ or 760 *μ*mol*·*mol^−1^ CO_2_ concentration, plant growth (three plants for each treatment) was assessed by regular (at 9:00 am every day) and nondestructive measurements of leaf length and leaf width (the third or fourth round of leaves), flower diameter, flower number, flower bud number, and lateral branch number for one month. Half of the product of leaf length multiplied by leaf width was regarded as the relative leaf area, and the mean of three times measurements of a flower's diameter from different angles was defined as the flower diameter. Flowers with fully unfolded corolla and buds with initial appearance of petals were included in the number of flower and flower bud, respectively. Leaf shape was quantified by means of bivariate allometric relationships between log-transferred leaf length and leaf width (*n* = 34), and the relationship between leaf length and leaf width determined the leaf shape of *Impatiens* New Guinea [12].

### 2.3. Determination of Photosynthetic Rate

Leaf Photosynthetic rate was measured with a Portable Photosynthesis system LI-6400 (LI-COR Inc., Lincoln, Neb, USA) after 45 days with three fully expanded leaves from each of five plants randomly selected from each treatment. The measurements for ambient CO_2_-grown plants were carried out at 1500 *μ*mol*·*m^−2^ *·*s^−1^ PAR, 2.0–2.5 KPa vapour pressure deficit (VPD), 23 ± 1°C and 380 *μ*mol*·*mol^−1^ CO_2_, and for elevated CO_2_-grown plants at 1500 *μ*mol*·*m^−2^ *·*s^−1^ PAR, 2.0–2.5 KPa VPD, 23 ± 1°C and 760 *μ*mol*·*mol^−1^ CO_2_.

### 2.4. Determination of Physiological and Biochemical Indexes

Leaves were obtained at the end of ten weeks' treatment from five plants of the control and elevated CO_2_-grown *Impatiens* New Guinea. Parts of the leaves were oven-dried at 60°C for the determination of starch and soluble sugars, and the other parts were stored in −80°C refrigerator following freezing in liquid nitrogen for the determination of contents of chlorophyll, MDA and activities of POD, SOD, APX. The content of starch was measured with the iodine colorimety method [14]. Chlorophyll, soluble sugars, MDA contents, and activities of POD, SOD were measured according to the methods of Wang [15]. The activity of APX was measured by method of Chen and Wang [16]. 

### 2.5. Leaf Ultrastructure Analysis

Areas beside the primary veins of fully expanded leaves were dissected into 1-2 mm^2^ squares and immediately fixed in 2.5% (v/v) glutaraldehyde (in 0.1 mol*·*L^−1^ phosphate buffer, pH 7.0) for 24 h. Then the samples were washed 5 times with the same buffer and postfixed in 1% osmium tetroxide for 3 h. After being washed with the same buffer for 3 times, leaf tissues were immediately passed through an ethanol dehydration series and then infiltrated and embedded in epoxy resin Epon-812. An ultramicrotome LKB-5 was used to cut sections. Thin sections were stained with uranyl acetate and lead citrate, and finally observations were carried out with a transmission electron microscope JEM-1200EX [9].

### 2.6. Statistical Analysis

The data are shown as mean ± standard deviation to indicate significant differences. Data were subjected to one-way analysis of variance and *t*-test using the SPSS software 16.0 (SPSS Inc, Chicago, Ill, USA). Morphological characteristics were determined based on 3 plants per sample, whereas physiological, chemical, and cellular characteristics were determined based on 5 plants per sample. All the analyses were repeated three times.

## 3. Results

### 3.1. Vegetative and Developmental Responses to Elevated CO_2_

**Figure 1:**
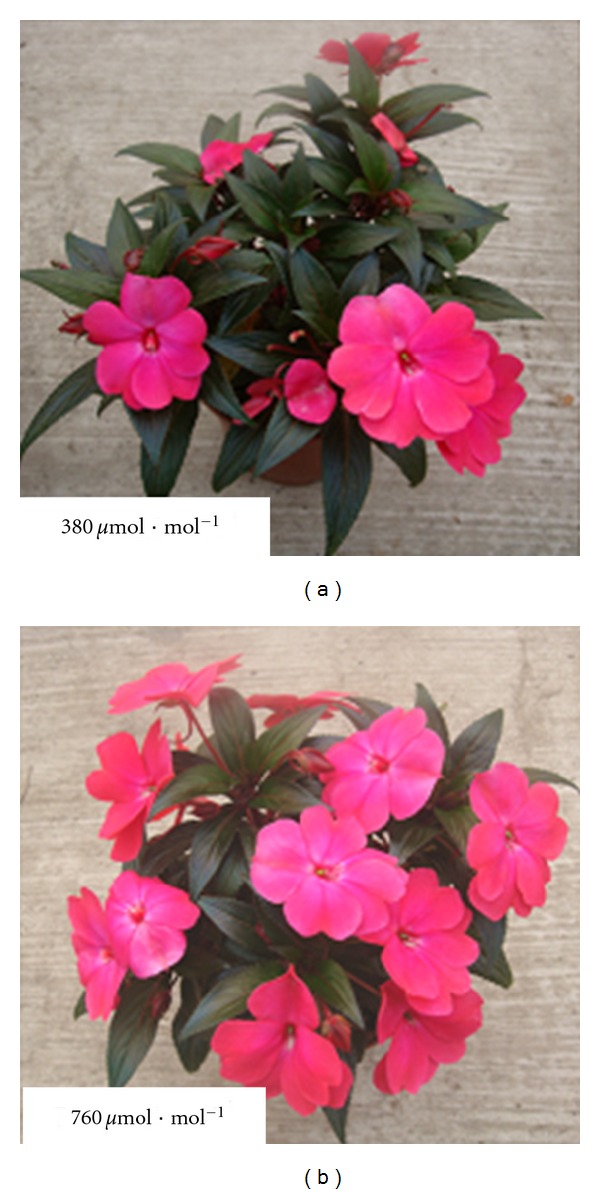
Growth situation of *Impatiens* New Guinea grown under two CO_2_ concentrations: ambient CO_2_ (380 *μ*mol*·*mol^−1^) and elevated CO_2_ (760 *μ*mol*·*mol^−1^) for 10 weeks.

**Figure 2:**
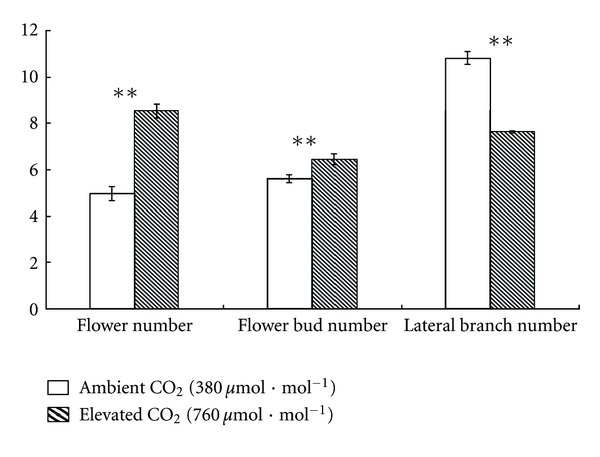
The numbers of flower, flower bud, and lateral branch of *Impatiens* New Guinea grown under two CO_2_ concentrations: ambient CO_2_ (380 *μ*mol*·*mol^−1^) and elevated CO_2_ (760 *μ*mol*·*mol^−1^). Asterisks show statistically significant differences (*P < 0.05; **P < 0.01; Student's *t*-test; *n* = 34 samples, with 3 plants per sample) and the bar is the standard deviation.

**Figure 3:**
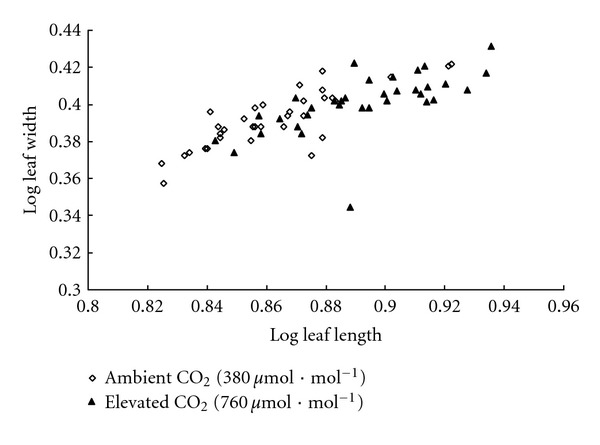
Allometric relationships between leaf length and leaf width for *Impatiens* New Guinea grown under two CO_2_ concentrations: ambient CO_2_ (380 *μ*mol*·*mol^−1^) and elevated CO_2_ (760 *μ*mol*·*mol^−1^). *n* = 34 samples, with 9 leaves from 3 plants per sample.

The *Impatiens* New Guinea used in this study displayed altered external features when subjected to elevated CO_2_ (Figures 1, 2, 3 and Table 1). The most conspicuous change of *Impatiens* New Guinea was the increased flower number per individual plant (Figures 1, 2), which was significantly enhanced by 72.18% (*P* < 0.0001), leading to higher visual value. Elevated CO_2_ also increased flower bud number per individual plant by 14.97% (Figure 2, *P* = 0.003). However, elevated CO_2_ had no significant effects on flower diameter (Table 1, *P* = 0.131). Besides, lateral branch number per individual plant was significantly reduced by 29.39% (Figure 2, *P* < 0.0001), thus inducing a relatively well-proportioned plant shape compared with plants in ambient CO_2_ which had more little lateral branches to occupy more space but seldom flowered (Figure 1).

Elevated CO_2_ also increased relative leaf area by 9.4% (Table 1, *P* < 0.001). The allometric relationships between leaf length and leaf width were analyzed to predict the changes of leaf shape (Figure 3). Log leaf length showed a 3.5% (Table 1, *P* < 0.0001) increase and log leaf width showed a 2.3% (Table 1, *P* = 0.009) increase, indicating that leaf length had a relatively bigger increase. Analyzing Table 1 and Figure 3 together, mean value of (log leaf length, log leaf width) of plants in ambient CO_2_ was (0.863, 0.392) and that of plants in elevated CO_2_ was (0.893, 0.401), showing that leaves in elevated CO_2_ appeared to be longer and wider than leaves in ambient CO_2_. Although the change of leaf shape seemed to be small, it was noticeable and measurable.

### 3.2. Responses of Photosynthetic Rate and Chlorophyll Content to Elevated CO_2_

Photosynthetic rate of *Impatiens* New Guinea grown in elevated CO_2_ was significantly accelerated compared with that of *Impatiens* New Guinea grown in ambient CO_2_ (Table 2), showing a 25.9% (*P* < 0.001) increase. However, total chlorophyll (chlorophyll a and chlorophyll b) content per unit leaf fresh weight of *Impatiens* New Guinea in elevated CO_2_ was significantly reduced (Table 2), showing an 18.2% (*P* < 0.001) decrease from that of *Impatiens* New Guinea in ambient CO_2_. Both chlorophyll a and chlorophyll b contributed to that response, which have a 26.2% and a 13.3% decrease, respectively, in spite of the indistinctive decrease of chlorophyll b (*P* = 0.15). The radio of chlorophyll a/b also dropped by 13.6% (Table 2, *P* = 0.004), indicating that the significant decrease of total chlorophyll mainly resulted from the decrease of chlorophyll a rather than chlorophyll b.

### 3.3. Responses of Nonstructural Carbohydrates to Elevated CO_2_

**Figure 4:**
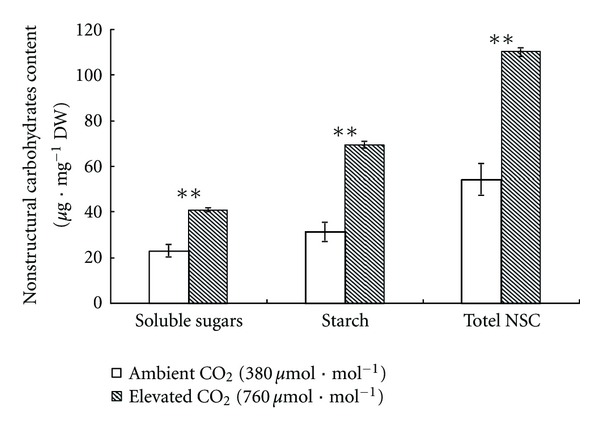
Nonstructural carbohydrates content of *Impatiens* New Guinea grown under two CO_2_ concentration: ambient CO_2_ (380 *μ*mol*·*mol^−1^) and elevated CO_2_ (760 *μ*mol*·*mol^−1^). Asterisks show statistically significant differences (*P < 0.05; **P < 0.01; Student's *t*-test; *n* = 3 samples, with 5 plants per sample) and the bar is the standard deviation. Total NSC= soluble sugars + starch.

Main nonstructural carbohydrates found in leaves are total soluble sugars and starch.  Figure 4 shows that elevation of CO_2_ had significant effect on nonstructural carbohydrates content per unit leaf dry weight of *Impatiens* New Guinea plants, which dramatically improved contents of soluble sugars and starch, showing a 77.81% (*P* < 0.001) and a 122.39% (*P* < 0.001) increase, respectively. Hence, the total nonstructural carbohydrates content of plants grown in elevated CO_2_ was doubled (a 103.5% increase, *P* < 0.001) compared with that of plants grown under ambient CO_2_.

### 3.4. Responses of Antioxidant Enzyme Activity and MDA Content to Elevated CO_2_

Elevated CO_2_ stimulated the activity of antioxidant enzymes, including peroxidase (POD), superoxide dismutase (SOD), and ascorbate peroxidase (APX) in these experiments of *Impatiens* New Guinea leaves (Table 3). The activity of POD, SOD, and APX increased by 119.78%, 11.01%, 73.26%, respectively, among which the increases of POD activity and APX activity reached significance level (*P* = 0.05), whereas the increase of SOD activity did not (*P* > 0.05). Besides, MDA content of *Impatiens* New Guinea leaves was remarkably reduced by 61.13% (*P* = 0.006) due to CO_2_ elevation (Table 3).

### 3.5. Responses of Leaf Ultrastructure to Elevated CO_2_

**Figure 5:**
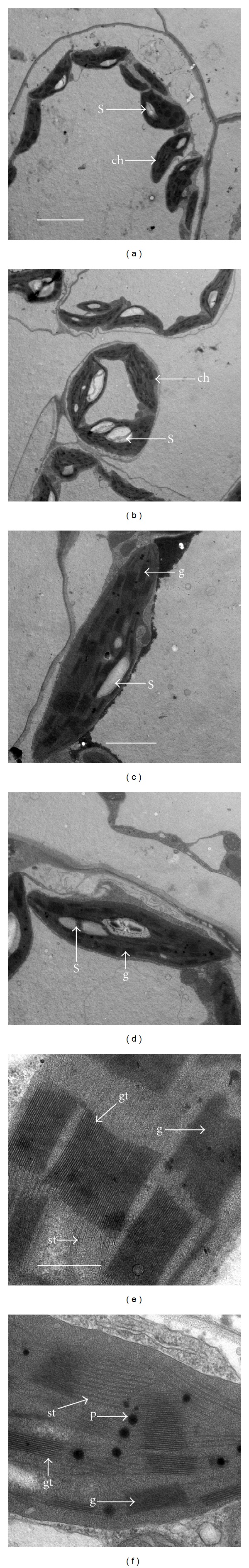
Transmission electron micrographs showing leaf ultrastructure of *Impatiens* New Guinea grown under two CO_2_ concentrations: ambient CO_2_ (a, c, e, 380 *μ*mol*·*mol^−1^) and elevated CO_2_ (b, d, f, 760 *μ*mol*·*mol^−1^). s: starch; ch: chloroplast; g: grana; gt: grana thylakoid; st: stroma thylakoids; p: plastoglobuli. Bars: 5 *μ*m (a and b); 2 *μ*m (c and d); 0.5 *μ*m (e and f).

Figures 5(a) and 5(b) show cross-sections through typical cells of *Impatiens* New Guinea leaves in ambient CO_2_ and elevated CO_2_, respectively. Chloroplasts were located peripherally and almost occupied half of the volume in the typical cell in elevated CO_2_ (Figure 5(b)). Starch grains were also observed, most of which are located at lateral sides of chloroplasts (Figures 5(a) and 5(b), arrows), leading to an ear-shaped protuberance arising from the surface. Figures 5(c) and 5(d) show that grana can be found throughout the chloroplast other than the volume occupied by starch grains. Figures 5(e) and 5(f) clearly show that grana were composed of numbers of grana thylakoids which were stacked orderly, making grana regular shape of cylinders. Moreover, stroma thylakoids were seen between grana (Figures 5(e) and 5(f), arrows), and plastoglobules were observed to spread in chloroplast around grana (Figure 5(f), arrows). 

As a consequence of elevated CO_2_, striking changes of the leaf ultrastructure were seen in the chloroplast. Firstly, chloroplasts showed an increased number of starch grains with expanded size (Figures 5(a), 5(b), 5(c), and 5(d)). Secondly, the number of grana in chloroplasts with elevated CO_2_ treatment seemed to be smaller than that of grana in ambient CO_2_ (Figures 5(c) and 5(d)), because of the greater number and size of starch grains. Furthermore, grana seemed to be reduced in size (Figures 5(e) and 5(f)), namely, the number of thylakoids making-up grana was reduced. However, elevated CO_2_ increased the number of stroma thylakoids between grana (Figures 5(e) and 5(f)), leading to an increased ratio of stroma thylakoid to grana thylakoid combining the opposite responses of stroma thylakoids and grana thylakoid numbers to CO_2_ elevation. Grana thylakoids in ambient CO_2_ were closely and orderly aligned (Figure 5(e)), whereas grana thylakoids in elevated CO_2_ were relatively loosely aligned (Figure 5(f)). This phenomenon was significantly observed in grana next to starch grains (Figures 5(c) and 5(d)). Finally, more plastoglobules seemed to be observed in chloroplasts with elevated CO_2_ treatment compared with those in ambient CO_2_ (Figures 5(e) and 5(f)).

## 4. Discussion

### 4.1. Morphological Characters

Flower number and flower bud number of *Impatiens* New Guinea were both significantly increased by elevated CO_2_. Similar results were found in other species such as Miniature rose, *Alstroemeria,* and *Lilium *[17–19]. Jablonski et al. [20] who used meta-analysis to integrate data from 159 enrichment papers providing information on 79 species found that growth at elevated CO_2_ resulted in the production of more (+19%) flowers. The significant stimulation of flower number and flower bud number of plants may primarily be a consequence of increased relative growth rate and accelerated developmental process which make plants reach the minimum size required for flowering earlier and have more resources available for reproduction, and finally increase flower number and flower bud number under elevated CO_2_ [9]. As flower number per individual plant is an important indicator for quality of ornamental plants, the remarkable enhancement of flower number and flower bud number by CO_2_ enrichment will be of great importance to improving the ornamental quality and market competitiveness of *Impatiens* New Guinea.

Besides, elevated CO_2_ also noteworthily increased relative leaf area of *Impatiens* New Guinea, that is, both leaf length and leaf width showed measurable increase, but leaf length had a relatively bigger increase. This is in accordance with many early reports [21, 22]. This increase of plant leaf area under elevated CO_2_ is probably an outcome of the well documented issue that elevated CO_2_ increased photosynthesis and carbohydrate production (discussed below). Ainsworth et al. [23] further demonstrated that at the transcript and metabolite level, CO_2_ enrichment stimulate the respiratory breakdown of carbohydrates, which provides increased energy and biochemical precursors for leaf expansion and growth, thus leading to increased leaf area of plants under elevated CO_2_. However, in a recent study, elevated CO_2_ had distinct effects on two *Aechmea* hybrids: *A. fasciata* “Maya” showed for both CO_2_ concentrations (380 *μ*mol*·*mol^−1^ and 750 *μ*mol*·*mol^−1^) an equal leaf area enhancement throughout the experimental period, whereas *A. fasciata* “Primera” showed a reduction of total leaf area by 41% [12]. A possible reason for this discrepancy is that the period of elevated CO_2_ treatment to this two *Aechmea* hybrids was too long (34 weeks), leading to the occurrence of CO_2_ acclimation, that is, the positive effects of long-term and high concentration CO_2_ treatments on plants will disappear gradually over time [6, 7].

### 4.2. Photosynthetic Rate, Chlorophyll Content, and Nonstructural Carbohydrates

In our study, photosynthetic rate of *Impatiens* New Guinea grown in elevated CO_2_ was significantly accelerated. This result was in accordance with many previous studies [8, 19, 21, 22], thus giving more evidence to the publicly accepted conclusion that CO_2_ enrichment is able to promote plant photosynthesis to some extent. However, in our study, total chlorophyll and chlorophyll per unit leaf fresh weight of *Impatiens* New Guinea in elevated CO_2_ was significantly reduced. We suppose that there is at least two reasons responsible for this result: firstly, the reduction of chlorophyll content may be caused by the dilution from excess accumulation of carbohydrates (discussed below); secondly, as Teng et al. [9] have proved of elevated CO_2_-induced reductions of mineral nutrient concentrations in plant leaves, including some of the basic components of chlorophyll such as N and Mg, so we hypothesize that the reductions of some necessary mineral nutrient may eventually affect the synthesis of chlorophyll.

A large number of studies have shown significant enhancement of carbohydrates (sugars and starches) in plant leaves by elevated CO_2_ [24–26]. It was reported that soluble sugars and starch contents increased by 50% and 160% on average, respectively [27]. We verified the previous studies with the result that contents of soluble sugars and starch of *Impatiens* New Guinea showed a 77.81% and a 122.39% increase, respectively, causing total nonstructural carbohydrates content doubled. We have documented above that elevated CO_2_ enhanced photosynthesis, which is in favor of the assimilation of nonstructural carbohydrates; in addition, limited sink capacity as well as some functional restriction such as limited phloem loading capacity and low efficiency of assimilate transport are likely to aggravate the accumulation of nonstructural carbohydrates in plant leaves under elevated CO_2_ [12].

However, with the increase of CO_2_ concentration or application time, plants tended to bring about the phenomenon of CO_2_ acclimation, which have been proved by many past studies to have a close relationship with these excess carbohydrates in plant leaves. Given the links between the phenomenon of CO_2_ acclimation and carbohydrates, it can be concluded as two aspects, one was that the excess accumulation of starch in plant leaves caused some physical damage to chloroplast ultrastructure (discussed below); the other was that soluble sugars suppressed photosynthesis by feedback inhibition [28].

### 4.3. Antioxidant Enzyme System

Antioxidant enzymes including peroxidase (POD), superoxide dismutase (SOD), and ascorbate peroxidase (APX) can neutralize free radicals, ridding the plant body of their harmful effects, therefore the activity of antioxidant enzymes is an important indicator of plant stress resistance [29]. Malonaldehyde (MDA) is a decomposition of peroxidized membrane lipid, so its content, which is closely related to the activity of antioxidant enzymes, can indicate the extent of membrane lipid peroxidation [30]. In our study, CO_2_ enrichment significantly improved activities of POD, SOD, and APX, but reduced MDA content, illustrating that antioxidant capacity of *Impatiens* New Guinea leaves was enhanced by elevated CO_2_. This phenomenon may be resulted from the significant stimulation of nonstructural carbohydrates in *Impatiens* New Guinea leaves (Figure 4), which can provide more energy substances for antioxidative metabolism, leading to improved activities of antioxidant enzymes and inhibited production of MDA, and eventually enhanced antioxidant capacity. Another possible interpretation for this is that elevated CO_2_ causes oxidative stress, thus signaling the need to increase the activity of antioxidant enzymes.

Because of the well-established link between antioxidant enzyme system and plant stress resistance, we suppose that stress resistance of *Impatiens* New Guinea would be strengthened. Several other plants have been reported with strengthened stress tolerance under elevated CO_2_ [31–35]. For instance, Sehmer et al. [31] found that under elevated CO_2_, the activities of SOD and APX of Norway spruce were enhanced when imposed to O_3_ stress to reduce the harm of O_3_ on its leaf tissue. Besides, as plant senescence is usually associated with the reduction of antioxidant enzymes activities [33], so we predict that elevated CO_2_ would delay the senescence of *Impatiens* New Guinea because of the significant enhancement of antioxidant activities. Rae et al. [34] proved at gene level that senescence was delayed under elevated CO_2_. In the investigation of Wang et al. [35] on effects of elevated CO_2_ on cut chrysanthemum, which were in great accordance with our findings, also found that elevated CO_2_ enhanced activities of POD and SOD, but reduced MDA content, therefore indirectly retarded cell degeneration and finally extended the life of cut flowers.

### 4.4. Leaf Ultrastructure

Many studies about effects of CO_2_ enrichment on plant leaf ultrastructure found that elevated CO_2_ increased the number and size of starch grains in chloroplast [6, 36, 37], which were consistent with our findings in biochemical assay of nonstructural carbohydrates (Figure 4) as well as leaf ultrastructure analysis (Figure 5) of* Impatiens* New Guinea. However, in an earlier study on young wheat leaves, more starch accumulation was observed in leaves in ambient CO_2_, whereas small starch grains were found to disperse throughout the stroma of chloroplasts in leaves grown under elevated CO_2_ [38]. A possible reason provided by the authors for this phenomenon was that during the early developmental stages of plants under elevated CO_2_, seedlings had a higher need for energy and carbon skeletons because of fast growth than those under ambient CO_2_, thus consuming more starch and leading to relatively less starch accumulated in young wheat leaves grown in elevated CO_2_.

In addition, elevated CO_2_ increased the ratio of stroma thylakoid to grana thylakoid, which agreed with many previous reports [9, 33, 39]. Griffin et al. [39] conjectured that these changes in the chloroplast structure maybe an approach to maintain leaf-level energy balance. They determined that, as elevated CO_2_ lead to a higher photosynthetic rate, increasing the demand for reductant to be used in carbon fixation and stroma thylakoids are enriched in photosystem I centers where NADPH is produced for reduction of CO_2_, so increased ratio of stroma thylakoid to grana thylakoid can ensure plant more efficiently fixation of CO_2_ into sugar products by increasing reductant production. Meanwhile, we found that grana thylakoids in ambient CO_2_ were closely and orderly aligned, whereas grana thylakoids in elevated CO_2_ were relatively loosely aligned, and this phenomenon was significantly observed in grana next to starch grains. There were several previous reports on elevated CO_2_-induced structural changes of thylakoids which were always accompanied with excess accumulation of starch grains [40, 41]. These findings indicated that structural changes of thylakoids may be caused by excess accumulation of starch grains which press against and separate grana thylakoids, thus changing the original close arrangement of grana thylakoids into loose. Taken together with elevated CO_2_-induced structural changes of thylakoids, these findings indicate that elevated CO_2_ damage the structure of leaf chloroplast to a certain extent, so we doubt whether the positive effects of elevated CO_2_ on growth and development of* Impatiens* New Guinea will continue or not with the increase of CO_2_ concentration or application time. Therefore, more work is needed to examine the detailed effects of higher concentration or long-term CO_2_ enrichment on growth and development of* Impatiens* New Guinea.

## 5. Conclusions

According to the above results, we are able to make a conclusion that CO_2_ enrichment has positive effects on *Impatiens* New Guinea, which can improve the visual value by increasing flower number and leaf area, promote growth by raising photosynthetic rate, nonstructural carbohydrates content, and ratio of stroma thylakoid to grana thylakoid, and enhance antioxidant capacity by improving activity of antioxidant enzymes. However, whether these positive effects of CO_2_ enrichment on *Impatiens* New Guinea will continue with the increase of CO_2_ concentration or application time still needs more investigations.

## Figures and Tables

**Table 1:** Leaf parameters and flower diameter of* Impatiens* New Guinea grown under two CO_2_ concentrations: ambient CO_2_ (380 *μ*mol*·*mol^−1^) and elevated CO_2_ (760 *μ*mol*·*mol^−1^).

	Ambient CO_2_ (380 *μ*mol*·*mol^−1^)	Elevated CO_2_ (760 *μ*mol*·*mol^−1^)	% increase	*P* value
Log leaf length	0.863 ± 0.004	0.893 ± 0.004	3.5	<0.001
Log leaf width	0.392 ± 0.003	0.401 ± 0.003	2.3	0.009
Relative leaf area (cm^2^)	9.016 ± 0.137	9.864 ± 0.140	9.4	<0.001
Flower diameter (cm)	6.615 ± 0.028	6.656 ± 0.023	0.6	0.131

Values given are mean ± standard deviation. Mean values (*n* = 34 samples, with 9 leaves or flowers from 3 plants per sample) were compared by Student's *t*-test.

**Table 2:** Chlorophyll content and photosynthetic rate of *Impatiens* New Guinea grown under two CO_2_ concentrations: ambient CO_2_ (380 *μ*mol*·*mol^−1^) and elevated CO_2_ (760 *μ*mol*·*mol^−1^).

	Ambient CO_2_ (380 *μ*mol*·*mol^−1^)	Elevated CO_2_ (760 *μ*mol*·*mol^−1^)	% increase	*P* value
Chlorophyll a (*μ*g*·*mg^−1^)	1.03 ± 0.02	0.76 ± 0.01	−26.2	<0.001
Chlorophyll b (*μ*g*·*mg^−1^)	0.45 ± 0.01	0.39 ± 0.01	−13.3	0.15
Total chlorophyll (*μ*g*·*mg^−1^)	1.81 ± 0.03	1.48 ± 0.02	−18.2	<0.001
Chlorophyll a/b radio	2.28 ± 0.02	1.97 ± 0.06	−13.6	0.004
Photosynthetic rate (*μ*mol*·*m^−2^ *·*s^−1^)	11.09 ± 0.30	13.96 ± 0.30	25.9	<0.001

Values given are mean ± standard deviation. Mean values (*n* = 3 samples, with 5 plants per sample) were compared by Student's *t*-test. Total chlorophyll: Chlorophyll a + Chlorophyll b. FW: fresh weight.

**Table 3:** Antioxidant enzyme activity and MDA content of *Impatiens* New Guinea grown under two CO_2_ concentration: ambient CO_2_ (380 *μ*mol*·*mol^−1^) and elevated CO_2_ (760 *μ*mol*·*mol^−1^).

	Ambient CO_2_ (380 *μ*mol*·*mol^−1^)	Elevated CO_2_ (760 *μ*mol*·*mol^−1^)	% increase	*P*-value
POD (U/(g∗min))	10.77 ± 2.83	23.67 ± 2.22	119.78	0.011
SOD (U/g)	527.75 ± 4.47	585.83 ± 39.51	11.01	0.141
APX ((U/(g∗min))	20.64 ± 0.88	35.76 ± 4.41	73.26	0.039
MDA (*μ*mol/g)	0.01114 ± 0.00142	0.00433 ± 0.00062	−61.13	0.006

Values given are mean ± standard deviation. Mean values (*n* = 3 samples, with 5 plants per sample) were compared by Student's *t*-test.
